# Pulpless Teeth and Alveolar Abscess

**Published:** 1877-08

**Authors:** S. C. Carter


### PULPLESS TEETH AND ALVEOLAR ABSCESS.

BY S. C. CARTER.

*Read before the Indiana State Dental Society.*

I regard the saving of pulpless teeth the most difficult and likely to be the most unsatisfactory service a dentist is called upon to perform, unless it is to insert artificial teeth on rubber or celluloid.

The saving or capping of exposed pulps is a far more satisfactory operation, and so often ends with that feeling of gratification a dentist is permitted sometimes to enjoy, in what is regarded by the people a most torturing business.

But a patient with a pulp-dying tooth possibly having suffered many days, and nursed the *offending member* many nights, and applied everything hot to his face and tooth he could find, from Radway's Ready Relief to a hot brick, and then sat for an hour at your office door in the vain hope of relief, when with a desperate effort and agony together, he enters your room hunted down at last, with fear, almost despair, depicted upon his countenance, he asks you to put something in his tooth to make it quit aching.

Then it is you most heartily wish there were no such folks, or they would call upon somebody else. For my part I prefer to serve people that will make every reasonable effort to avoid such a condition of their teeth.

The quickest and most successful treatment of such teeth, of two or more roots with abscess formed or forming, is to carefully separate with a sharp instrument the gum from the neck of the tooth well down to the alveolus, with a parallel slit terminating at the buccal, bifurcation of the roots or both buccal and lingual if lower teeth.

If cold weather, warm your iron, and place it well down on the tooth being careful to not grip too tightly, and crush the tooth. Give the necessary outward and inward movement very steadily and firmly, and only far enough to perceive you have moved the tooth in these directions, and have certainly loosened it. Then carefully lift it from its socket.

To preserve the tooth a long time, keep it dry. The decay will be arrested. The abscess cured.

I do not wish the learned members of this Association, in their kindness and disposition to treat their weaker and more ignorant brethren with so much charity, to think I have not attempted to treat a pulpless tooth, or alveolar abscess with a view to retain the tooth in use.

On the contrary I have, according to my books, since March 15th, 1876, filled the roots of 58 teeth.

As far as I know every one is in service. Though abscess formed or commenced to form in two instances after dismissal, which I successfully treated.

Of the pulpless teeth thus filled, twenty-six were molars, and thirty-two (to be short) were not molars.

I have treated since the time named twenty-eight cases of alveolar abscess with a purpose to save the teeth, and kept twenty-two in service as far as I know.

These teeth have come under my notice in three conditions as follows:

*First Condition.*—Pulp dead, and had been for some sometime and the issue at the apex thoroughly healed, tooth otherwise healthy, except decay in crown.

*Second Condition.*—Pulp almost gone, or in a state of decomposition, yet showing sensibility in the roots. Patient suffering little or no inconvenience from it, but the tooth is ready for trouble first time the patient is severely exposed or health impaired.

*Third Condition.*—Abscess formed or forming, and the patient suffering, and sometimes suffering the extremes of torture possible from a tooth.

TREATMENT.

*Condition First.*—I immediately prepare and fill the roots, and crown and dismiss the case.

I operated upon fourteen of the fifty-eight teeth in this way. It was two of these fourteen teeth that abscess formed or commenced to form after dismissal.

I am therefore of the opinion I was mistaken in believing the nerves at the point of the roots were cicatrized.

*Condition Second.*—I regard the treatment of teeth in this condition as a critical time, both for patient and dentist. To the patient for the reason already stated. "The tooth is ready for trouble first time the patient is seriously exposed, or health impaired, or tooth improperly treated.

To the dentist as he risks receiving the credit of making a tooth ache.

The object is to bring these to a healthy condition first.

To examine these teeth I have brought to view the pulp cavity with its canal openings, keeping the *debris* washed away with warm water, or blown away if using the Rubber Dam.

I then take a pulp extractor, and endeavor to remove the pulp in the roots if pain is not too great. I at least remove all possible under the circumstances, I avoid enlarging the canal, as I think the risk of irreparable damage or accidents too great to be compensated by anything that may be gained thereby, and particularly so, if the canals are crooked or flat, or both.

At every call of the patient I attempt to remove the pulp, until I am satisfied it is all away; after each effort to remove the pulp I force carbolic acid into the canals usually by taking a refused nerve extractor with enough barbes on it to hold the proper amount of cotton wound around it, to pump or force the acid into the canals. Then insert an easily removed temporary filling cautioning the patient to take care of health and avoid exposure as that tooth will ache if not very careful in this particular.

In a few cases I have had patients to return with toothache, and abscess forming. The tooth going into Condition Third instead of Condition First as desired. In most instances, however, I have succeded in time. Sometimes two or three months in bringing such teeth into Condition First.

I have of late used *Salicylic* acid very sucessfully on teeth in this condition.

The idea I received from reading Dr. Taft's Operative Dentistry.

The plan I have adopted, is to force all the powered acid I can into the canal, and often fill the pulp cavity with same, then insert a temporary filling, and dismiss the patient for two months.

I believe in every case I have thus used *Salicylic* acid, I found the tooth after two or three months reduced to Condition First.

*Condition Third.*—There is only one pleasant thought for the dentist in finding a tooth in this condition, and that is, "He cannot easily make it worse."

'Tis true after wading through much torture for your patient, you may receive his thanks, and half dollars enough to pay you for your trouble, and time, and leave the tooth in his mouth. But after a tooth has tortured him so, he will always be afraid of it, and associate you with that particular tooth, and possibly let other teeth suffer because he abhors you, and your profession: will tell you he will "let them all rot out, and get a new set, and would rather have all his teeth pulled out than one filled." The very sight or thought of you will start a cold chill.

Now to be short I am as often disposed to deliver these teeth to the forceps as I am to treat them unless it is a front tooth.

On the account of soreness and nervousness of patient, these teeth are difficult to "open up."

The pulp a half alive and dead mass; often alive enough to cause too much pain to remove it, and I have applied arnica. After removing all I can of the pulp, and cleansing the cavity with warm water I use again the everlasting carbolic acid. Cause the gum around the tooth to bleed freely. Saturate a pledget of cotton with any excellent biting counter irritant and apply to the buccal or label gums about even with points of the roots, and sometimes hold a pledget of same for a while to the lingual or pallatal gum, have painted these teeth and surrounding gums with iodine.

My patient is apt to be temporarily out of health otherways. I have noticed that sudden unfavorable changes of weather mature a crop of these teeth. And I usually call the attention of my patient to his health, and recommend him to care for and improve it.

If I can get the irritation reduced, and the tooth to Condition First it is all I can expect.

But, sometimes these abscesses form openings other than through the tooth. After an opening is once formed I expect final sucess by keeping the tooth and abscess cleansed and injecting carbolic acid a time or two. Then Salicylic acid as I regard the tooth in Condition Second.

It is very unfortunate, however, when the abscess is allowed to point outwardly through the face or cheek, as I have seen in two cases. (Not my own however.)

An unsuccessful attempt had been made to extract these teeth crushing the crown allowing the roots to remain.

In cases where the direction of the abscess cannot be otherwise controlled and is pointing to an unfortunate direction, I believe I would attempt an artificial opening through the alveolus. However, I have never been so unfortunate as to resort to this operation, therefore cannot speak from experience.

The *dennier resort* is to extract the tooth. If you fail to do that with all these accumulated woes upon you, sit down by your patient and mourn with him.

After pulpless teeth are reduced to Condition First, I fill the roots with Os. Artificial.

I endeavor to measure the length of the canal. It is here you want to be a good guesser. I then do my best to guess its length. I then take a fiber of cotton of a size to carry as much of the thinly mixed Os. Artificial as I think can be easily forced up to stop the further end of the canal, and slowly force it to the desired place allowing the air to pass out. Then take another fiber, load it, send it along not quite so far. Then fill the canal and after that the pulp chamber.

